# Supplementary material for: The Effect of Antibiotics on the Infant Gut Fungal Microbiota
Source: J Fungi (Basel). 2022 Mar 22;8(4):328. doi: 10.3390/jof8040328 (PMC9032081; doi:10.3390/jof8040328)
Supplement: Supplementary file 1 [file jof-08-00328-s001.zip › SupplementaryFigures_110222.pptx]

## Slide 1
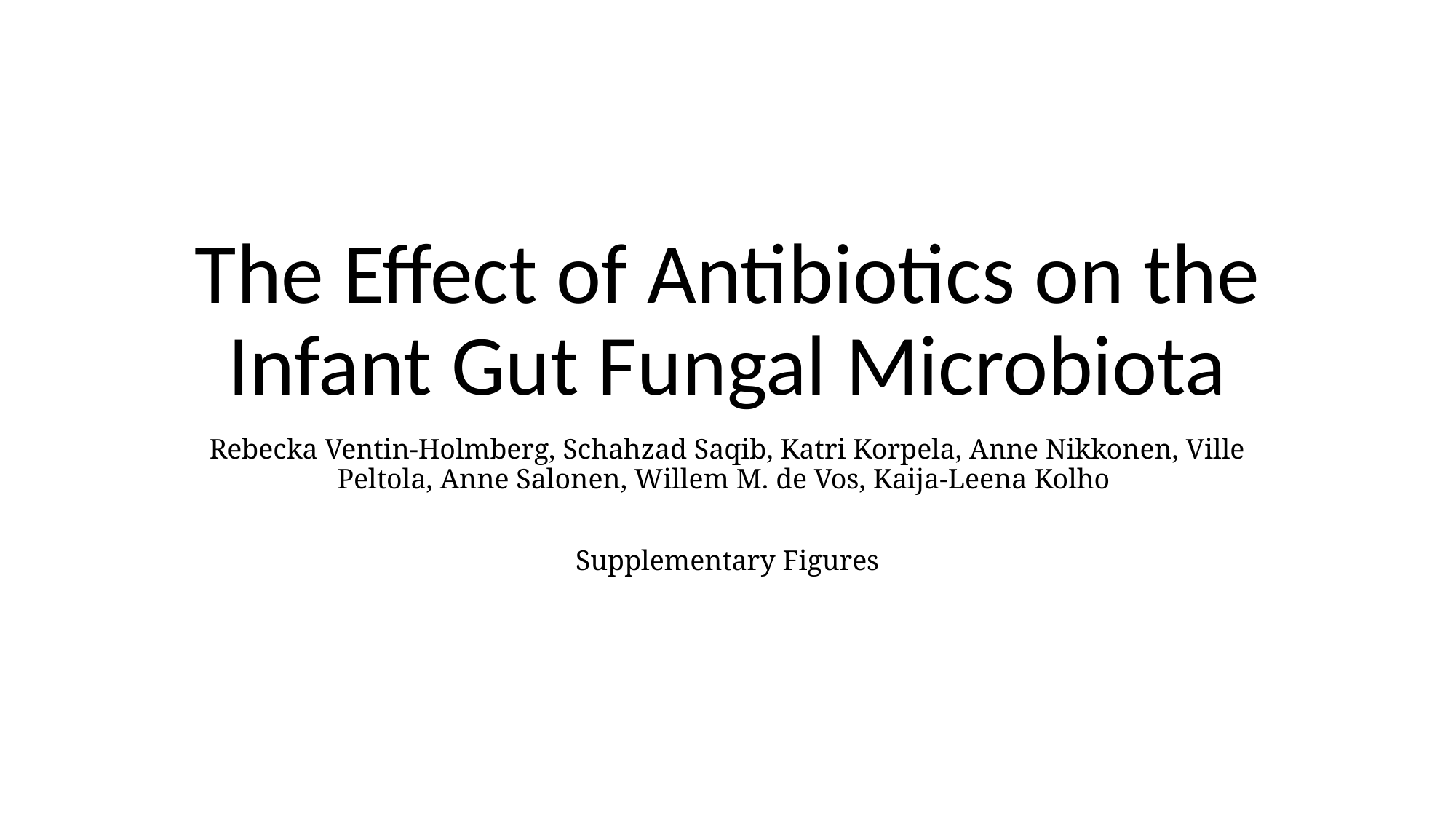

# The Effect of Antibiotics on the Infant Gut Fungal Microbiota
Rebecka Ventin-Holmberg, Schahzad Saqib, Katri Korpela, Anne Nikkonen, Ville Peltola, Anne Salonen, Willem M. de Vos, Kaija-Leena Kolho
Supplementary Figures

## Slide 2
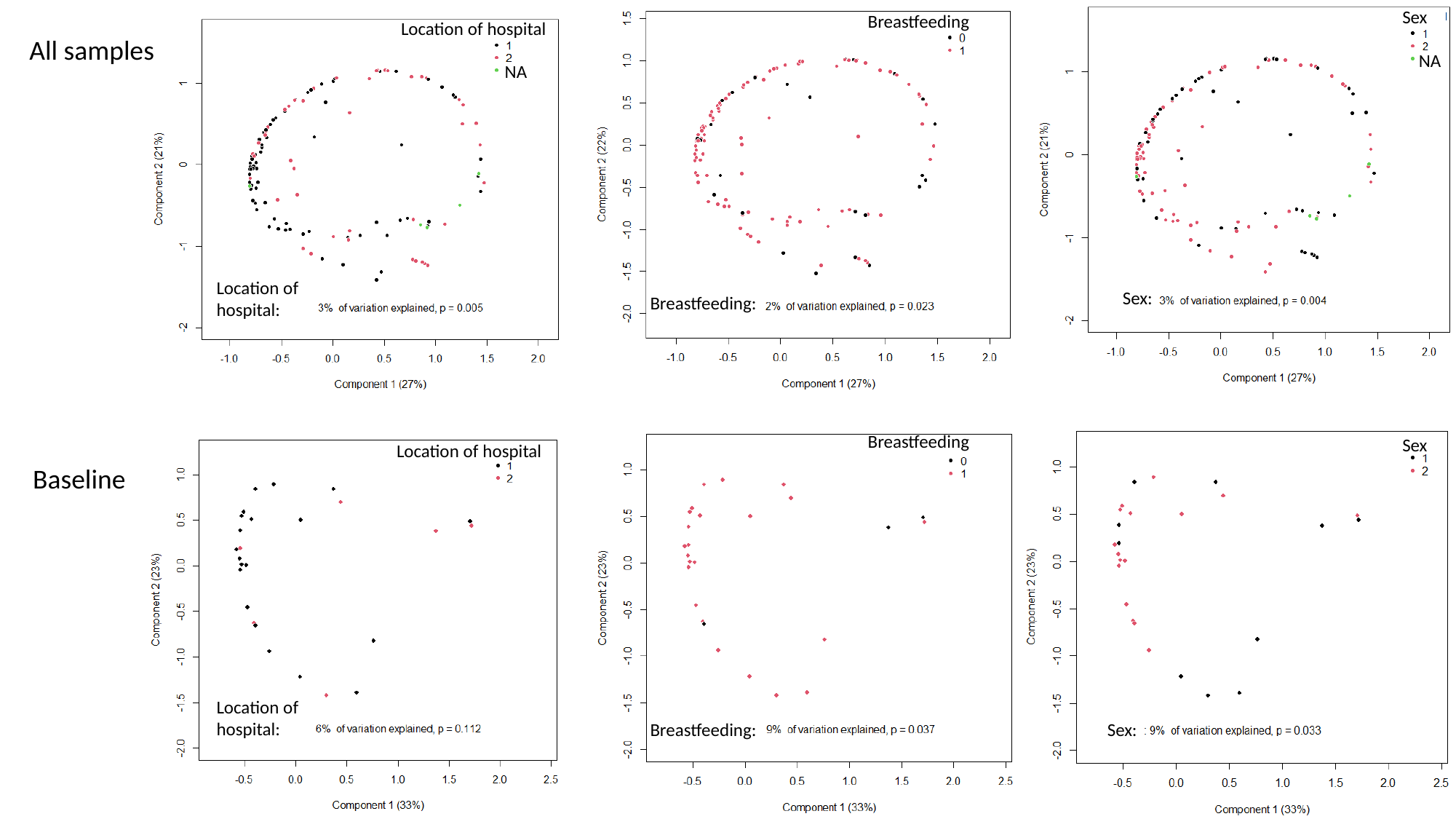

Sex
Breastfeeding
Location of hospital
All samples
NA
NA
Location of
hospital:
Sex:
Breastfeeding:
Breastfeeding
Sex
Location of hospital
Baseline
Location of
hospital:
Sex:
Breastfeeding:

## Slide 3
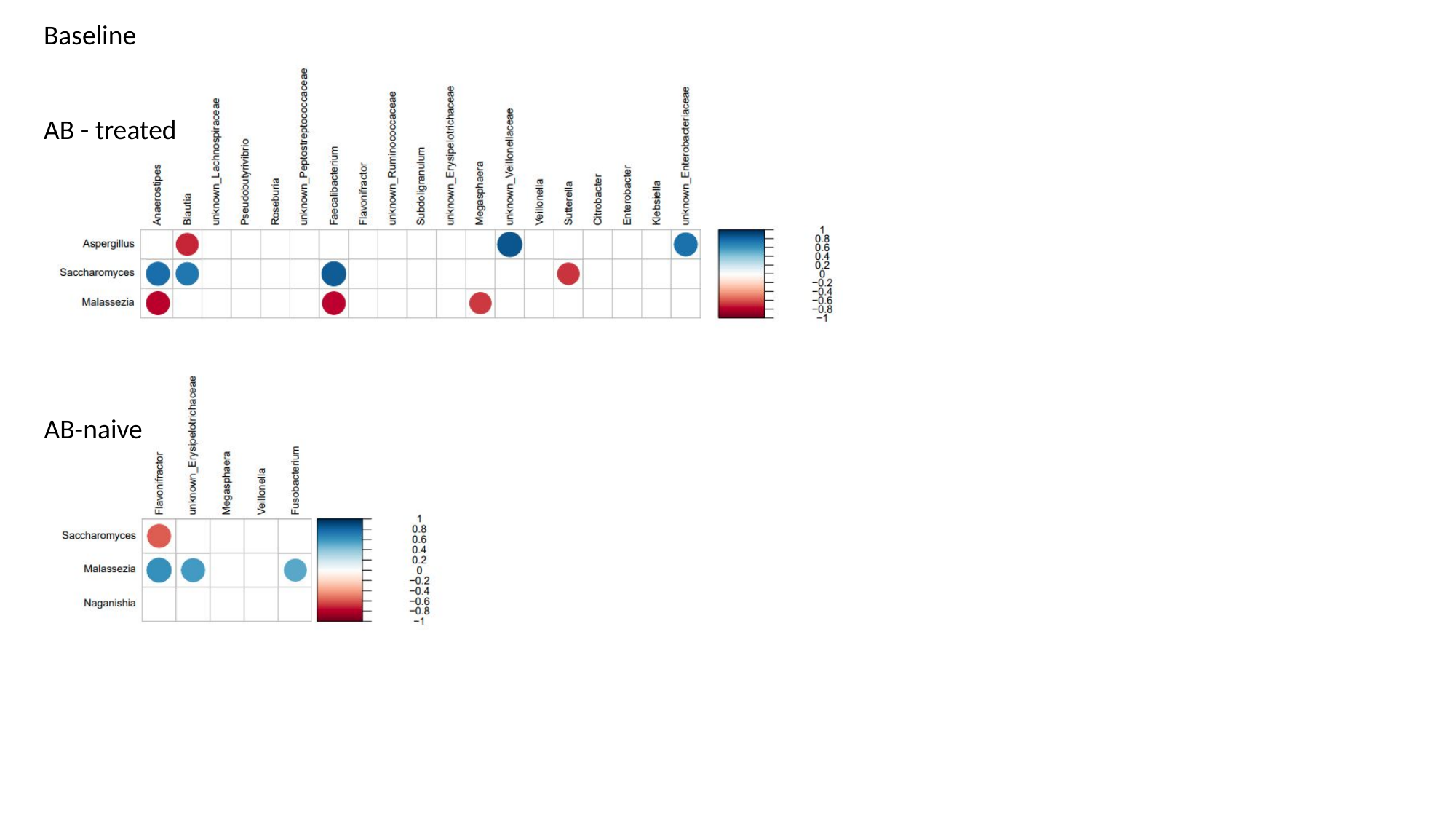

Baseline
AB - treated
AB-naive

## Slide 4
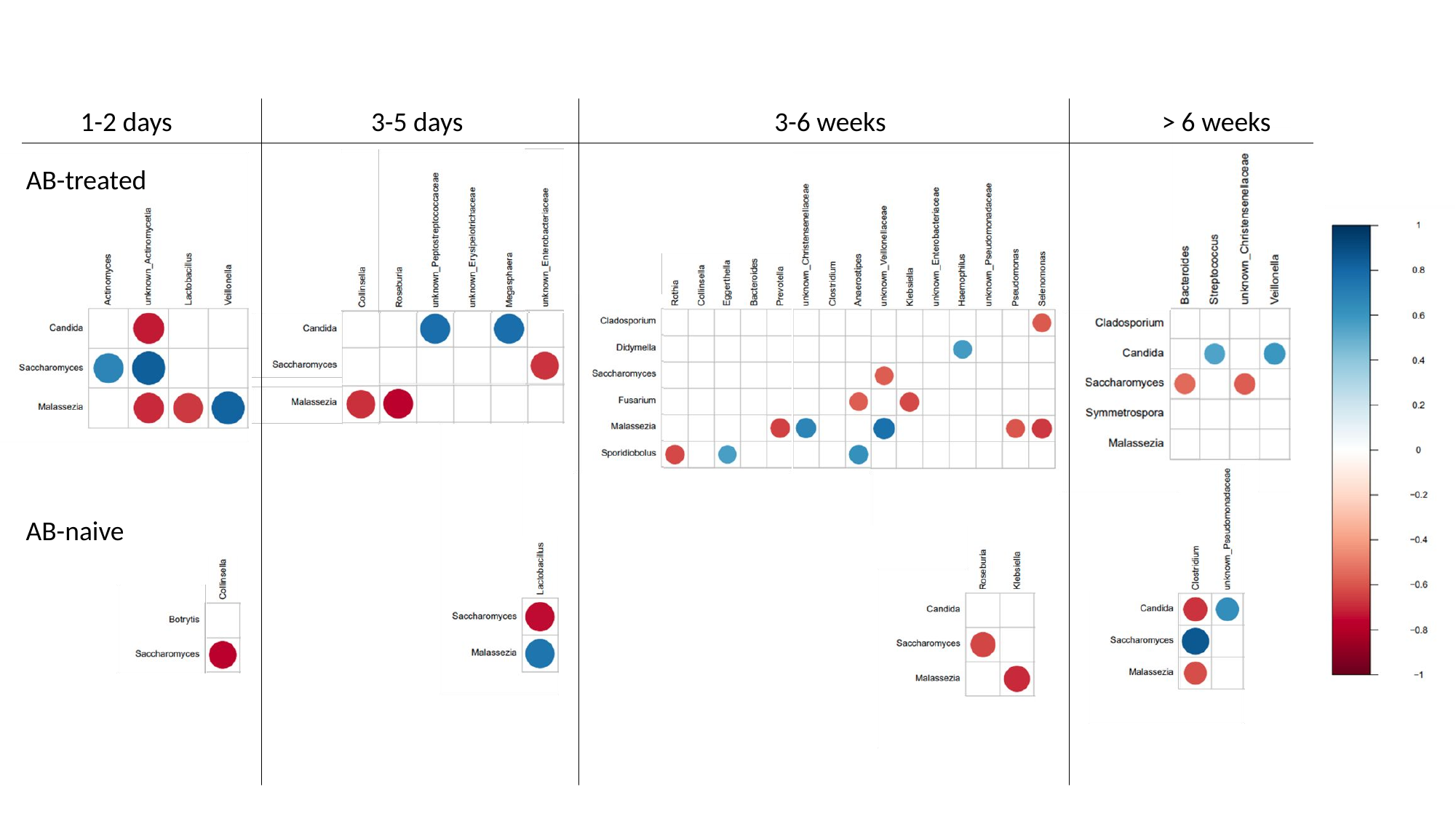

1-2 days
3-5 days
3-6 weeks
> 6 weeks
AB-treated
AB-naive

## Slide 5
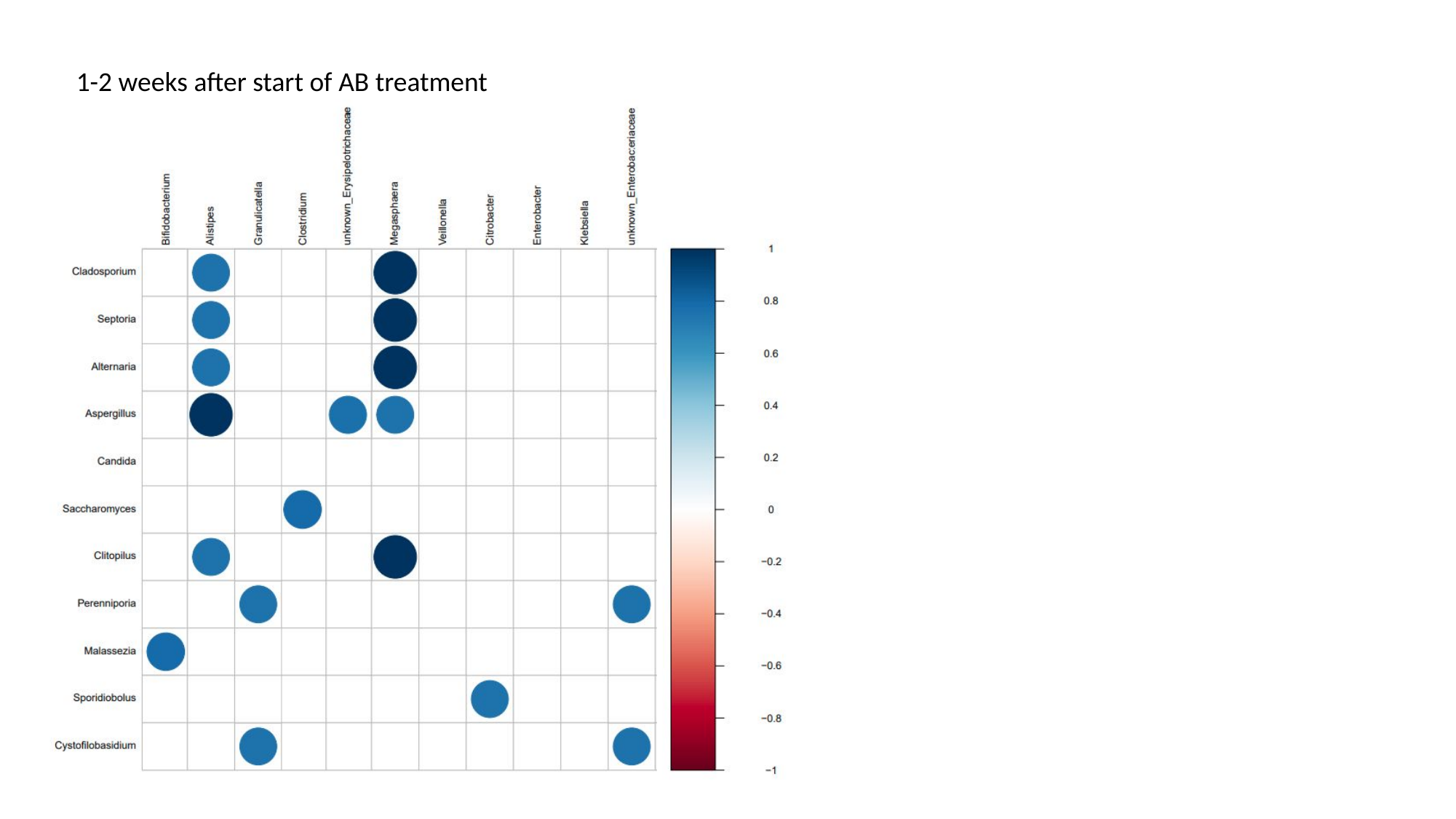

1-2 weeks after start of AB treatment
